# Acoustic signals in the sand fly *Lutzomyia (Nyssomyia) intermedia *(Diptera: Psychodidae)

**DOI:** 10.1186/1756-3305-4-76

**Published:** 2011-05-13

**Authors:** Felipe M Vigoder, Nataly A Souza, Alexandre A Peixoto

**Affiliations:** 1 Laboratório de Biologia Molecular de Insetos, Instituto Oswaldo Cruz, FIOCRUZ, Av. Brasil 4365, Manguinhos, CEP 21040-360, Rio de Janeiro, RJ, Brazil; 2 Laboratório de Transmissores de Leishmanioses, Instituto Oswaldo Cruz, FIOCRUZ, Av. Brasil 4365, Manguinhos, CEP 21040-360, Rio de Janeiro, RJ, Brazil; 3 Instituto Nacional de Ciência e Tecnologia em Entomologia Molecular, Brazil

## Abstract

**Background:**

Acoustic signals are part of the courtship of many insects and they often act as species-specific signals that are important in the reproductive isolation of closely related species. Here we report the courtship songs of the sand fly *Lutzomyia (Nyssomyia) intermedia*, one of the main vectors of cutaneous leishmaniasis in Brazil.

**Findings:**

Recordings were performed using insects from three localities from Eastern Brazil: Posse and Jacarepaguá in Rio de Janeiro State and Corte de Pedra in Bahia State. The three areas have remnants of the Brazilian Atlantic forest, they are endemic for cutaneous leishmaniasis and *L. intermedia *is the predominant sand fly species. We observed that during courtship *L. intermedia *males from all populations produced pulse songs consisting of short trains. No significant differences in song parameters were observed between the males of the three localities.

**Conclusions:**

*L. intermedia *males produce acoustic signals as reported for some other sand flies such as the sibling species of the *Lutzomyia longipalpis *complex. The lack of differences between the males from the three localities is consistent with previous molecular studies of the *period *gene carried out in the same populations, reinforcing the idea that *L. intermedia *is not a species complex in the studied areas and that the three populations are likely to have similar vectorial capacities.

## Findings

Understanding sexual behavior of insect vectors is important as it can be a possible target for disease control [1,2]. Acoustic signals in intraspecific communication of insects is frequently associated with mating [3,4] and they often act as species-specific signals either for long range attraction, e.g. crickets [5], or in close range recognition and stimulation, e.g. fruit flies [6].

Many vector species also produce songs as part of their mating behavior [7-9]. In the sand fly *Lutzomyia longipalpis s.l.*, main vector of American visceral leishmaniasis, the male lovesong has proven to be a good marker to distinguish the Brazilian cryptic species of this complex [10-12]. Songs have also been reported from two other Neotropical sand fly species: *Lutzomyia cruzi *and *Lutzomyia migonei *[13,14].

*Lutzomyia (Nyssomyia) intermedia, or Nyssomyia intermedia *[15], is one of the main vectors of cutaneous leishmaniasis in Brazil [16]. Two other species very closely related to *L. intermedia *are also important vectors of cutaneous leishmaniasis, *Lutzomyia neivai *and *Lutzomyia whitmani *[16-18]. The first one has a more limited distribution being found in more southern regions of Brazil and northern Argentina, while the second one has a larger distribution and is found in sympatry with *L. intermedia *and *L. neivai *in many regions [17].

Despite its epidemiological importance very little is known about the sexual behavior of *L. intermedia*. Here we report the songs produced by males of *L. intermedia *from three different populations from Eastern Brazil.

Insects used in the present study were collected in the localities of Posse (Petrópolis, Rio de Janeiro State), Jacarepaguá (Rio de Janeiro, Rio de Janeiro State) and Corte de Pedra (Presidente Tancredo Neves, Bahia State). The three areas have remnants of the Brazilian Atlantic forest, they are endemic for cutaneous leishmaniasis and *L. intermedia *is the predominant sand fly species.

The sand flies were identified according to Young & Duncan [19] and Marcondes [17] and the F1 of wild-caught females were used in the experiments. Sand flies were raise in constant temperature (25°C ± 1°C) with humidity close to saturation using the same protocol used for *L. longipalpis *[20].

The recordings of the acoustic signals were also carried out at 25°C ± 1°C using an INSECTAVOX microphone [21], a Sony Hi8CCD-TRV65 video camera and Sony SLV-77HFBR VCR with a virgin couple of the same population in each trial as described in Souza et al. [11]. Each recording lasted about 5 minutes and male wing vibration towards the female was observed in about 20% of the trials. Although no copulation was observed in any of the trials, this song appears to be part of the courtship as the males start to sing when they are facing the female's side moving simultaneously towards her back. The recorded songs were digitalized using a CED1401 A/D converter and the analysis was performed using the Spike2 software (version 4.08), both from Cambridge Electronic Design (UK). Four song parameters were analyzed from each song: inter-pulse-interval (IPI) and train length (TL) measured in milliseconds (ms) and seconds (s), respectively, number of pulses per train (NP) and carrier frequency (FREQ) in Hertz (Hz). The statistics analysis was performed using the software R [22].

**Figure 1:**
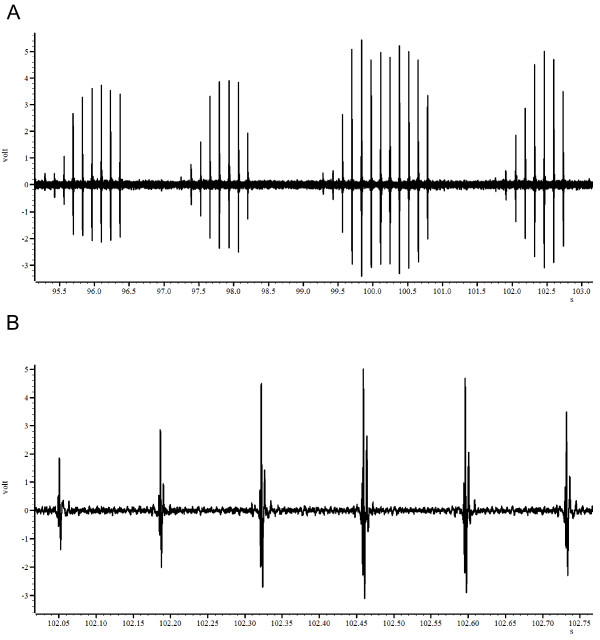
***Lutzomyia intermedia *male song**. Panel (A) shows a segment of 8 seconds of song with 4 trains. Panel (B) shows a single train in more detail.

*L. intermedia *males produce short trains of pulse song (Figure 1). The means (± SEM) of the parameters analyzed in the three *L. intermedia *populations are shown in Table 1. Individuals from the population of Posse presented the higher IPI and the shorter trains while males from Corte de Pedra showed the higher song frequency. However, ANOVA indicates no significant difference in any of the parameters analyzed (IPI: F_2,21 _= 2.88, NP: F_2,21 _= 2.02; TL: F_2,21 _= 1.21; Freq: F_2,21 _= 0.50; CPP: F_2,21 _= 0.45; p > 0.05 in all cases).

**Table 1:** Mean values (± SEM) for the song parameters from three populations of *L. intermedia*.

	N	IPI (ms)	NP	TL (s)	Freq (Hz)	CPP
**Jacarepaguá**	3	130.12 (± 3.43)	11.67 (± 0.33)	1.39 (± 0.01)	230.04 (± 4.34)	2.50 (± 0.16)

**C. de Pedra**	10	138.65 (± 2.21)	10.41 (± 0.60)	1.30 (± 0.08)	249.68 (± 16.48)	2.72 (± 0.08)

**Posse**	9	142.88 (± 3.16)	8.80 (± 1.01)	1.11 (± 0.14)	232.38 (± 10.97)	2.62 (± 0.17)

Compared to the songs produced during copulation by *L. migonei *males [14], the (pre-copulatory) courtship song of *L. intermedia *has a much larger IPI (~137 ms × ~26 ms), much longer trains (~1.3 s × ~0.15 s) and smaller CPP (~2.6 × ~3.3). The song of *L. intermedia *males also contrasts with the copulation songs produced by the different *L. longipalpis *siblings species [10-12], even those producing pulse song which are characterized by shorter IPIs (< 67 ms) and longer trains (> 2.1 s). Songs produced before and during copulation have probably the same basic function; they are part of the courtship, increasing female's receptivity to insemination and having also a role in reproductive isolation of closely related species.

Since no copulations were observed we cannot be sure the song is really involved in courtship. Another possibility is that it is involved in some form of aggression, such as to protect a territory. However, this behavior is usually a male-male signal [3] and no songs were produced in a couple of trials where two males were used together with a female. Therefore, it seems more likely that songs are part of courtship.

The lack of mating prevented us from verifying whether *L. intermedia *males also produce copulation songs as observed in *L. longipalpis s.l*., *L. cruzi *and *L. migonei *[11-14]. In Drosophila, although males of some species sing while in copula [23], in most studied species males only produce courtship songs before attempting copulation [6]. Therefore, although it is possible that *L. intermedia *males also produce copulation songs that is not necessarily the case.

In *L. longipalpis s.l*. populations the analysis of male lovesongs and the *period *gene, which is involved in the control of Drosophila courtship song [24], yielded consistent results confirming the existence of a species complex in Brazil [10-12,25,26].

The analysis of the differentiation in the *period *gene between the *L. intermedia *populations of Posse, Jacarepaguá and Corte de Pedra [18] is also consistent with the song results as the two types of analysis did not indicate significant differentiation, either molecular or behavioral. Therefore, these data together reinforce the idea that, at least for the studied localities, *L. intermedia *constitutes a single species and that the three populations are likely to have similar vectorial capacities.

## Competing interest

The authors declare that they have no competing interests.

## Authors' contributions

FMV participated in the recordings, performed the song and statistical analysis and drafted the manuscript. NAS collected the insects and participated in the recordings. AAP conceived the study, participated in the recordings and helped to write the manuscript. All authors have read and approved the final manuscript.
